# Supplementary material for: Natural and Artificial Sweeteners and High Fat Diet Modify Differential Taste Receptors, Insulin, and TLR4-Mediated Inflammatory Pathways in Adipose Tissues of Rats
Source: Nutrients. 2019 Apr 19;11(4):880. doi: 10.3390/nu11040880 (PMC6520815; doi:10.3390/nu11040880)
Supplement: Supplementary file 1 [file nutrients-11-00880-s001.pdf]

Table S1: Primers used in real time PCR.

Gen	Forward sequence (5'-3')	Reverse sequence (5'-3')
rgip	GTTCCTTCCAGGGTTTCCTC	GACCTCATCTCCAGGCACAT
tlr-4	GTGCCCCGCTTTCAGCTTTG	GTGCCTCCCCAGAGCATTGT
tnf-α	ATGTGGAACTGGCAGAGGAG	GCCATGGAACTGATGAGAGG
glut-4	GGGCTGTGAGTGAGTGCTTTC	CAGCGAGGCAAGGCTAGA
srebp-1c	CGTTGTACTGCAGCCACACT	AGTGGTACTGTGGCCAGGAT
pparγ	CGACCTGGAAGTCCAACTAC	ATCTGCTGCATCTGCTTG
adiponectin	GCCTCTACGATACGGTCCAA	TGCATTCTGACCTTCACCAC
leptin	TTTCACACACGCAGTCGGTATC	GGTCTGGTCCATCTTGGACAAA
ucp-1	GCCTCTACGATACGGTCCAA	TGCATTCTGACCTTCACCAC
bcat2	AGGCACTGCTTGTCAGGTCT	CTCCCTAACGTCACACACGA
bckdh	AAGGAGCGTCACTTCGTCAC	AGAGGTTGGTGTGGAGATGG
36b4	CGACCTGGAAGTCCAACTAC	ATCTGCTGCATCTGCTTG
cyclophilin	CGTGGGCTCCGTTGTCTT	TGACTTTAGGTCCCTTCTTCTTATCG
